# Supplementary material for: Identification of Bioactive Compounds from Marine Natural Products and Exploration of Structure-Activity Relationships (SAR)
Source: Antibiotics (Basel). 2021 Mar 22;10(3):337. doi: 10.3390/antibiotics10030337 (PMC8004798; doi:10.3390/antibiotics10030337)
Supplement: Supplementary file 1 [file antibiotics-10-00337-s001.pdf]

Contents

1. Summary of HPLC Methods 2

2. HPLC Fractionation and Purification 3

3. Mass Spectrometry Data for Natural Products 8

4. Nuclear Magnetic Resonance Spectroscopy Data for natural products 19

HPLC FRACTIONATION AND PURIFICATION

1.1 Summary of HPLC Methods.

Table S1 Summary of HPLC methods for the purification of key samples of interest.

AIMS Sample Code	Source Information	HPLC Step 1	HPLC Step 2
**19033**	Phylum: *Porifera*<br>Class: *Demospongiae*<br>Order: *Dendroceratida*<br>Family: *Dysideidae*<br>Genus: *Dysidea*	Reversed-Phase (C18), 1% TFA in H_2O/MeCN, 60 min 0-100% MeCN, then 100% for 10 min, 1 mL min^{-1}	Reversed-Phase (C18), 1% TFA in H_2O/MeCN, 20 min 0-40% MeCN,60 min 40-100% MeCN, 10 min 100% MeCN, 7 mL min^{-1}
**20608**	Phylum: *Porifera*<br>Class: *Demospongiae*<br>Order: *Dictyoceratida*<br>Family: *Spongiidae*<br>Genus: *Lendenfeldia*	Reversed-Phase (C18), 1% TFA in H_2O/MeCN, 60 min 0-100% MeCN, then 100% for 10 min, 1 mL min^{-1}	Reversed-Phase (C18), 1% TFA in H_2O/MeCN, 20 min 0-40% MeCN, 60 min 40-100% MeCN, 10 min 100% MeCN, 7 mL min^{-1}
**26104**	Phylum: *Porifera*<br>Class: *Demospongiae*<br>Order: *Dictyoceratida*<br>Family: *Irciniidae*<br>Genus: *Ircinia*	Reversed-Phase (C18), 1% TFA in H_2O/MeCN, 60 min 0-100% MeCN, then 100% for 10 min, 1 mL min^{-1}	Reversed-Phase (C18), 1% TFA in H_2O/MeCN, 20 min 0-40% MeCN, 60 min 40-100% MeCN, 10 min 100% MeCN, 7 mL min^{-1}
**25663**	Phylum: *Chordata*<br>Class: *Ascidiacea*<br>Order: *Flavobranchia*	Reversed-Phase (C18), 1% TFA in H_2O/MeCN, 60 min 0-100% MeCN, then 100% for 10 min, 1 mL min^{-1}	Reversed-Phase (C18), 1% TFA in H2O/MeCN, 60 min 0-40% MeCN, 30 min 40-100% MeCN, 10 min 100% MeCN, 7 mL min^{-1}
**26051**	Phylum: *Porifera*<br>Class: *Demospongiae*<br>Order: *Dictyoceratida*<br>Family: *Irciniidae*<br>Genus: *Ircinia*	Reversed-Phase (C18), 1% TFA in H_2O/MeCN, 60 min 0-100% MeCN, then 100% for 10 min, 1 mL min^{-1}	Reversed-Phase (C18), 1% TFA in H_2O/MeCN, 20 min 0-40% MeCN, 60 min 40-100% MeCN, 10 min 100% MeCN, 7 mL min^{-1}

**25641**	Phylum: *Porifera*<br>Class: *Demospongiae*<br>Order: *Dictyoceratida*<br>Family: *Spongiidae*,<br>Genus: *Spongia*	Reversed-Phase (C18), 1% TFA in H_2O/MeCN, 60 min 0-100% MeCN, then 100% for 10 min, 1 mL min^{-1}	Reversed-Phase (C18), 1% TFA in H_2O/MeCN, 20 min 0-40% MeCN, 60 min 40-100% MeCN, 10 min 100% MeCN, 7 mL min^{-1}

1.2 Fractionation of AIMS Sample 19033.

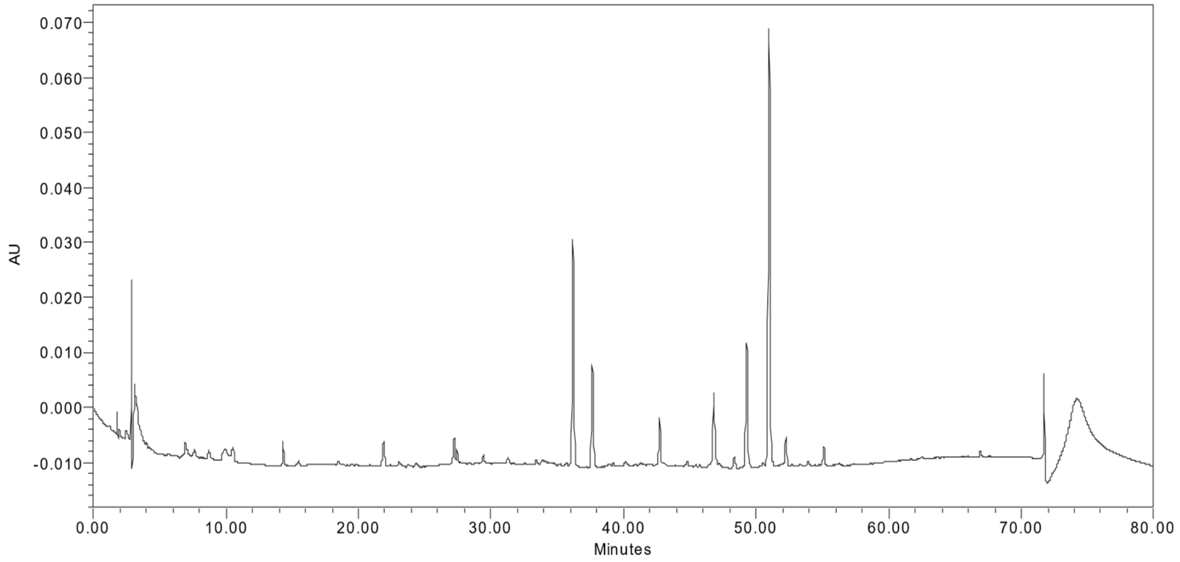

Figures S1 Representative HPLC trace for sample 19033. Preparative HPLC was carried out using a C18 RP-HPLC column and a gradient with 0 to 100 % acetonitrile-H_2O, flow rate 1 mL min^{-1}, monitored at 254 nm, to yield 80 fractions over 80 minutes.

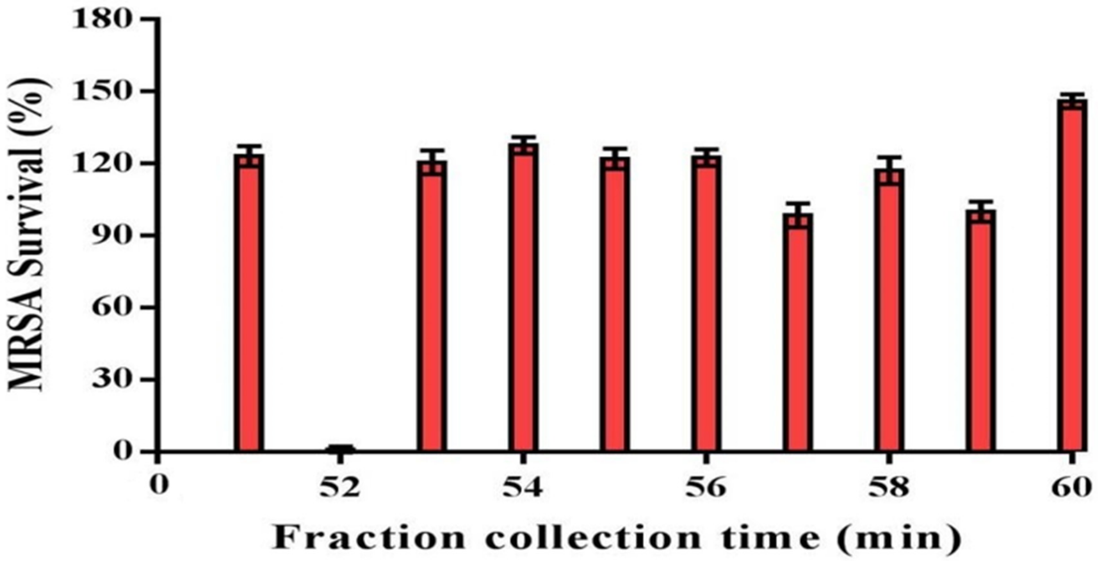

Figures S2 Bioassay of selected fractions from HPLC purification of sample 19033. Fractions were tested for bioactivity against MRSA. Data show mean survival ± SEM of duplicate samples.

1.3 Fractionation of AIMS Sample 20608.

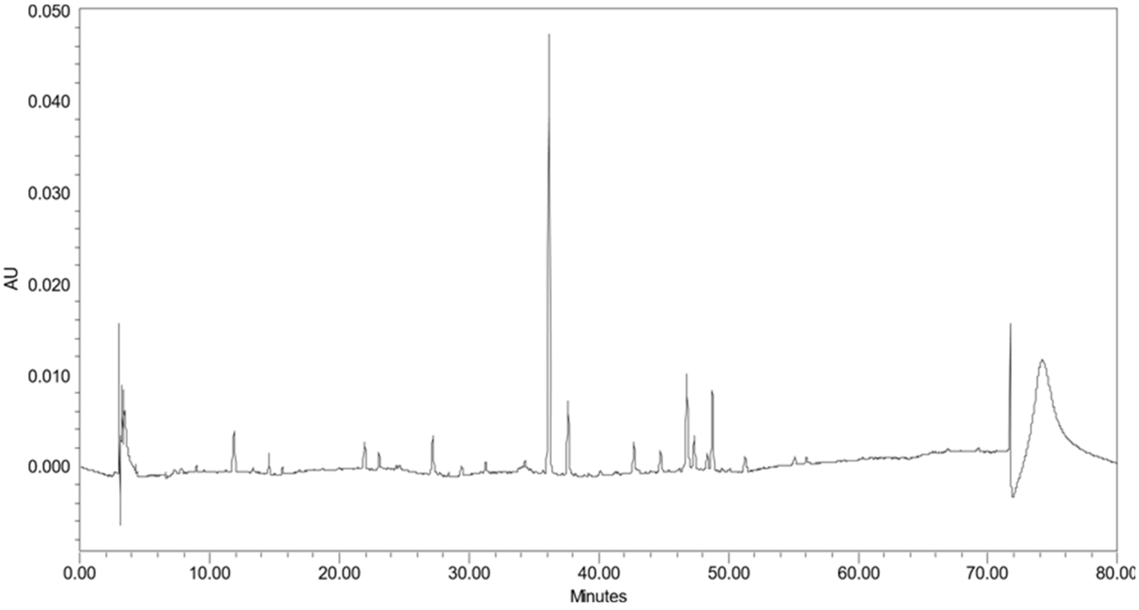

Figures S3 Representative HPLC trace for sample 20608. Preparative HPLC was carried out using a C18 RP-HPLC column and a gradient with 0 to 100 acetonitrile-H_2O, flow rate 1 mL min^{-1}, monitored at 254 nm, to yield 80 fractions over 80 minutes.

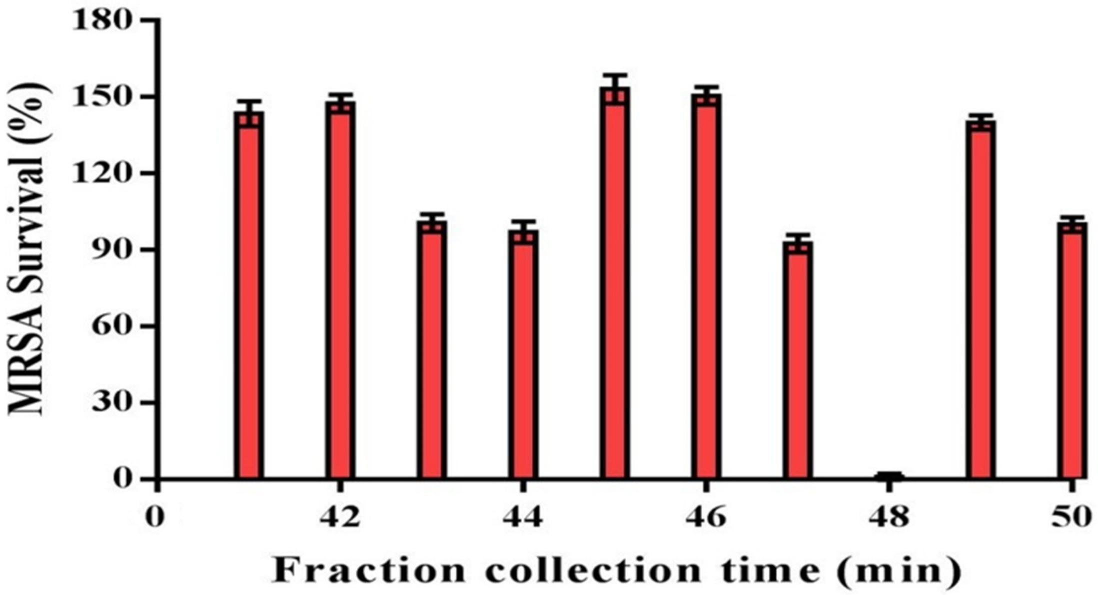

Figures S4 Bioassay of selected fractions from HPLC purification of sample 20608. Fractions were tested for bioactivity against MRSA. Data show mean survival ± SEM of duplicate samples.

1.4 Fractionation of AIMS Sample 26051.

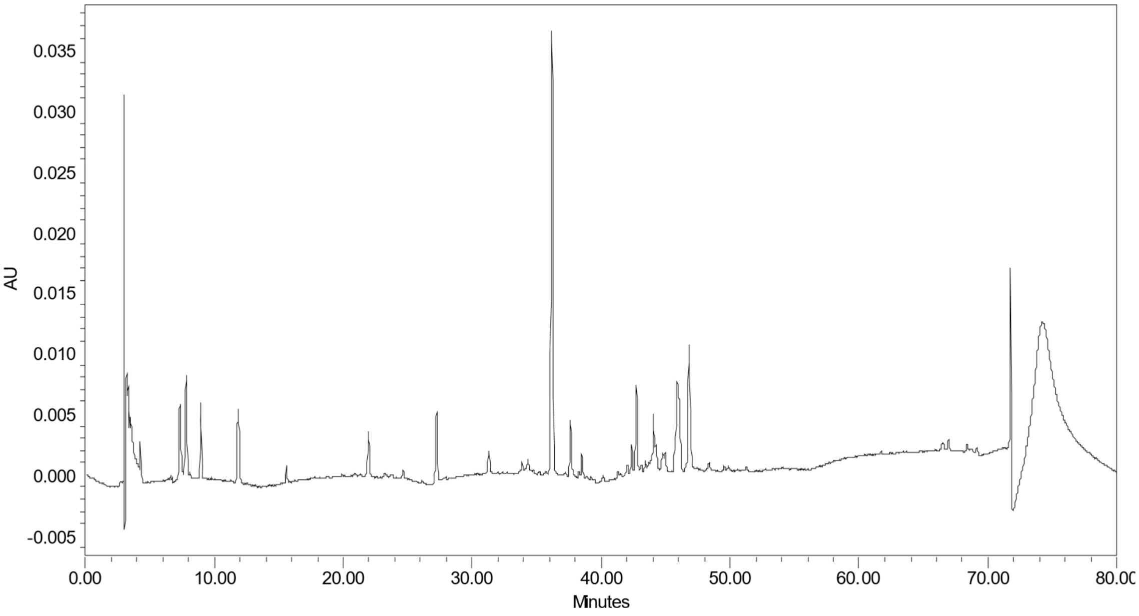

Figures S5 Representative HPLC trace for sample 26051. Preparative HPLC was carried out using a C18 RP-HPLC column and a gradient with 0 to 100 acetonitrile-H_2O, flow rate 1 mL min^{-1}, monitored at 254 nm, to yield 80 fractions over 80 minutes.

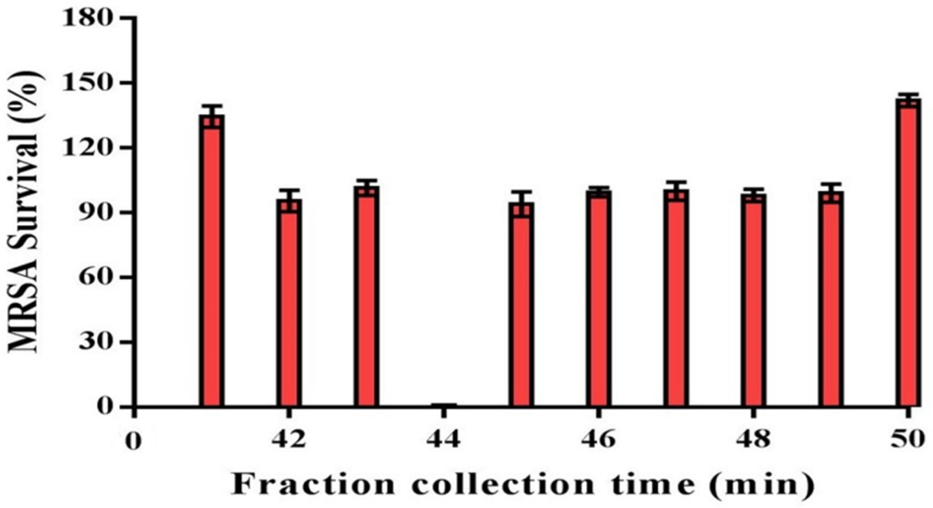

Figures S6 Bioassay of selected fractions from HPLC purification of sample 26051. Fractions were tested for bioactivity against MRSA. Data show mean survival ± SEM of duplicate samples.

1.5 Fractionation of AIMS Sample 25663.

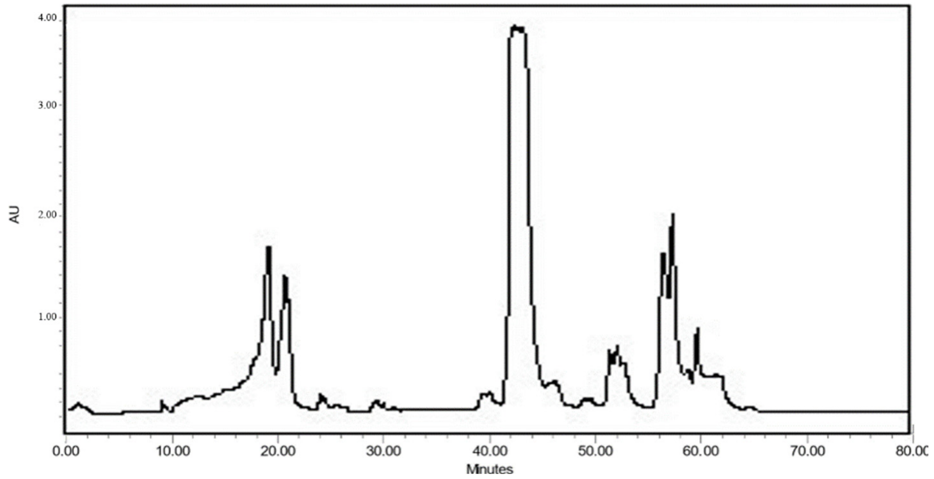

Figures S7 HPLC of sample 25663. Preparative HPLC was carried out using a C18 RP-HPLC column and a gradient with 0 to 100 acetonitrile-H_2O, flow rate 1 mL min^{-1}, monitored at 254 nm, to yield 80 fractions over 80 minutes.

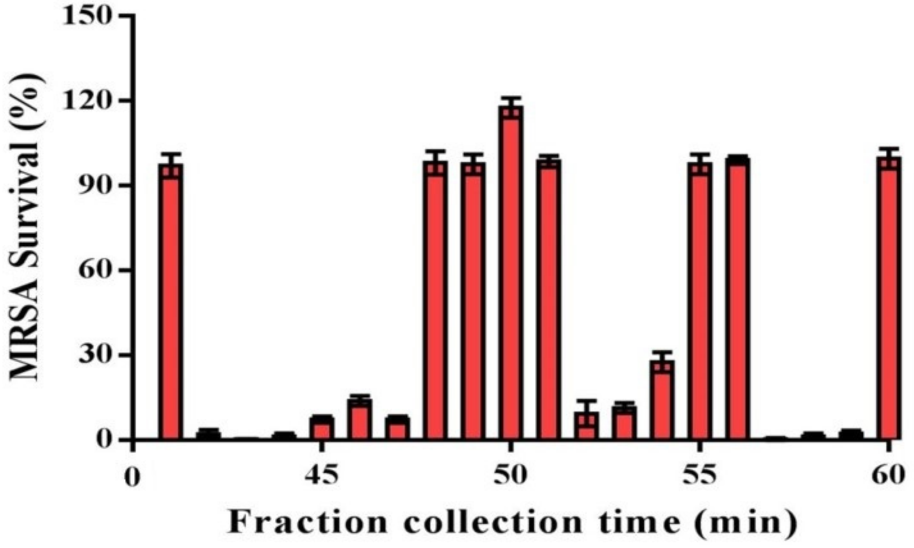

Figures S8 Bioassay of selected fractions from HPLC purification of sample 25663. Fractions were tested for bioactivity against MRSA. Data show mean survival ± SEM of duplicate samples.

1.6 Fractionation of AIMS Sample 26104.

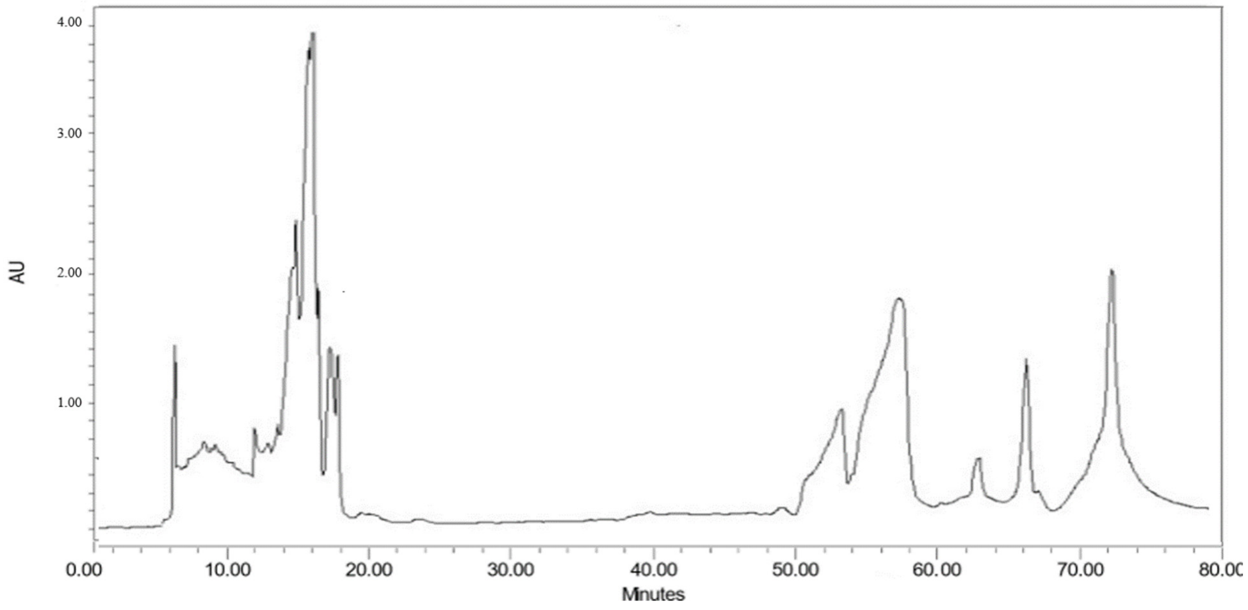

Figures S9 HPLC of sample 26104. Preparative HPLC was carried out using a C18 RP-HPLC column and a gradient with 0 to 100 acetonitrile-H_2O, flow rate 1 mL min^{-1}, monitored at 254 nm, to yield 80 fractions over 80 minutes.

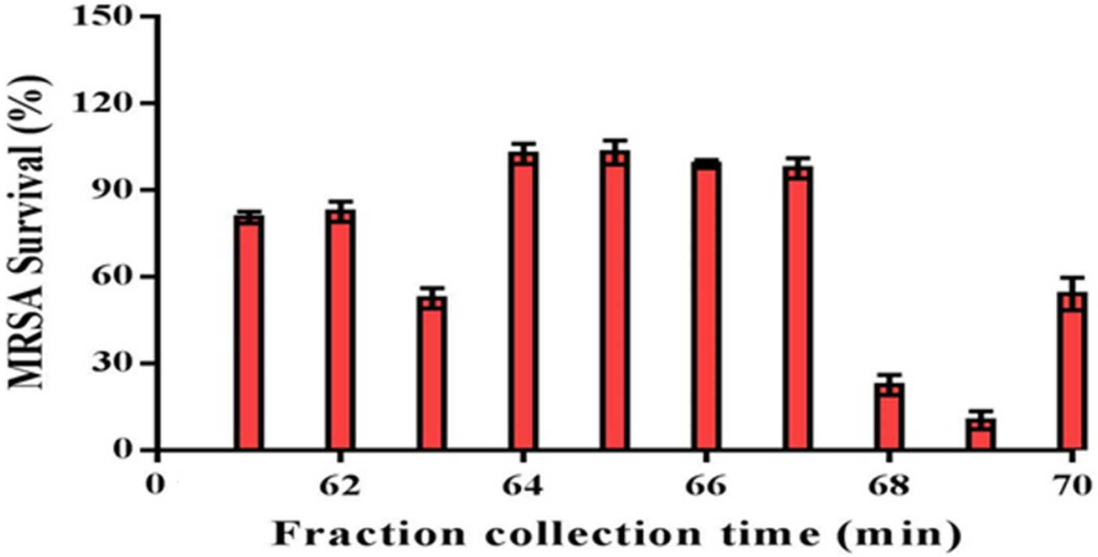

Figures S10 Bioassay of HPLC fractions from sample 26104. Fractions were tested against MRSA for isolation bioactive part. Data shows mean survival ± SEM of duplicate samples.

1. MASS SPECTROMETRY DATA FOR NATURAL PRODUCTS.

2.1. MS Analysis of AIMS Sample 19033.

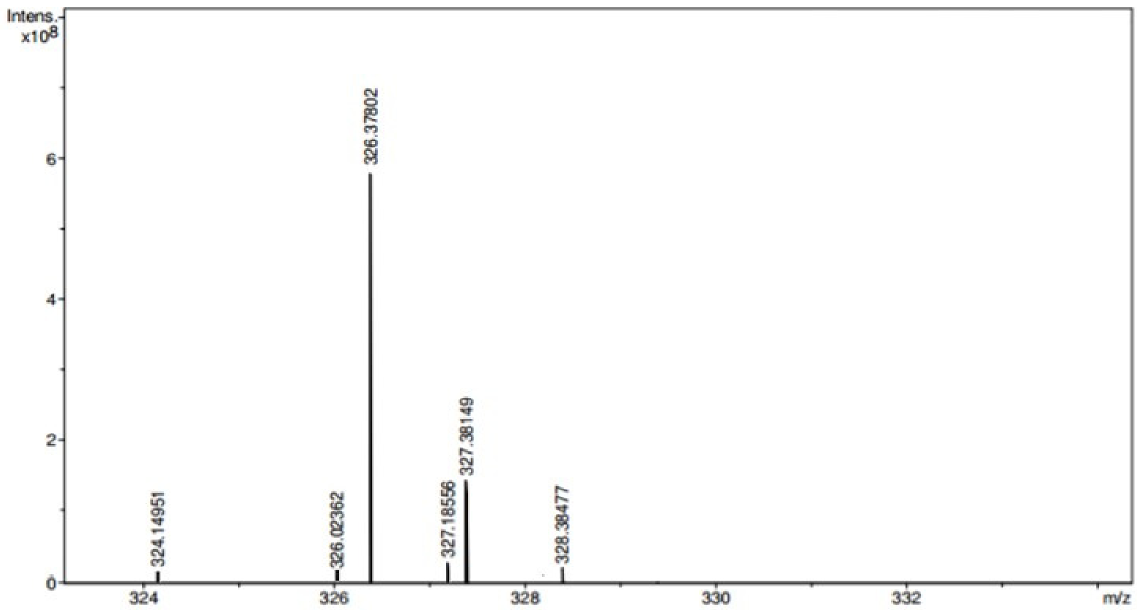

Figures S11 HRMS spectrum of the biologically active component of extract 19033.

MS analysis of the bioactive component of sample number 19033 shows a molecular ion at experimental *m/z* 326.37813; assuming $[CHNO+Na_{0-1}]^+$ theoretical *m/z* for $[C_{22}H_{48}N]^+$ = 326.37802; Δm = 0.11 ppm, RDBE = 0 (Figures S11). The MS/MS fragmentation shows fragments consistent with saturated hydrocarbon chains, up to C_{10} in length, leading to the proposal of the tertiary amine structure **3** for this compound. Key daughter ions and suggested fragment structures are represented in Table S2 and fragmentation pathways in Figures S12.

Table S2 MS/MS, MS^3 and MS^4 data and assignments for AIMS sample 19033.

Parent ion peak	MS/MS or MS^3 pattern	Neutral loss (*m/z* or amu)	Daughter ion peak	Fragment form	Predictive structure
**326 $[C_{22}H_{48}N+H]^+$ (rdbe 0)**	→ 324	[M-2H]	324	$[C_{22}H_{46}N]^+$	Double bond or ring formation
	→ 241	($C_{15}H_{39}N$) or ($C_{14}H_{37}$)	107	$[C_7H_9N]^+$ or $[C_8H_{11}]^+$	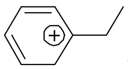 or 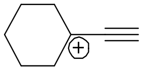
		($C_{12}H_{23}$)	159	$[C_{10}H_{25}]^+$	C_{10} hydrocarbon chain
	→ 241→159	($C_{17}H_{43}N$) or ($C_{18}H_{45}$)	65	$[C_5H_5]^+$ or $[C_4H_3N]^+$	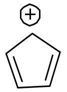 or 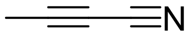
	→ 243	(C_6H_{11})	243	$[C_{16}H_{37}N]^+$	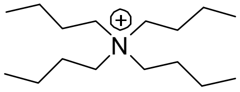
	→ 186	($C_{18}H_{39}N$) or ($C_{19}H_{37}$)	57	$[C_4H_9]^+$ or $[C_3H_7N]^+$	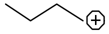 or 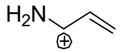
		($C_{17}H_{37}N$) or ($C_{18}H_{39}$)	71	$[C_5H_{11}]^+$	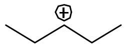
		($C_{16}H_{35}N$) or ($C_{17}H_{37}$)	85	$[C_6H_{13}]^+$ or $[C_5H_{11}N]^+$	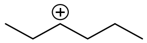
		($C_{15}H_{33}N$) or ($C_{16}H_{35}$)	99	$[C_7H_{15}]^+$ or $[C_6H_{13}N]^+$	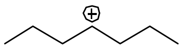
	→ 186 → 85	($C_{18}H_{39}N$) or ($C_{19}H_{41}$)	57	$[C_4H_9]^+$ or $[C_3H_7N]^+$	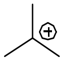
		($C_{18}H_{41}N$) or ($C_{19}H_{43}$)	55	$[C_4H_7]^+$ or $[C_3H_5N]^+$	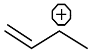

RDBE (rdbe) = ring or double bond equivalents

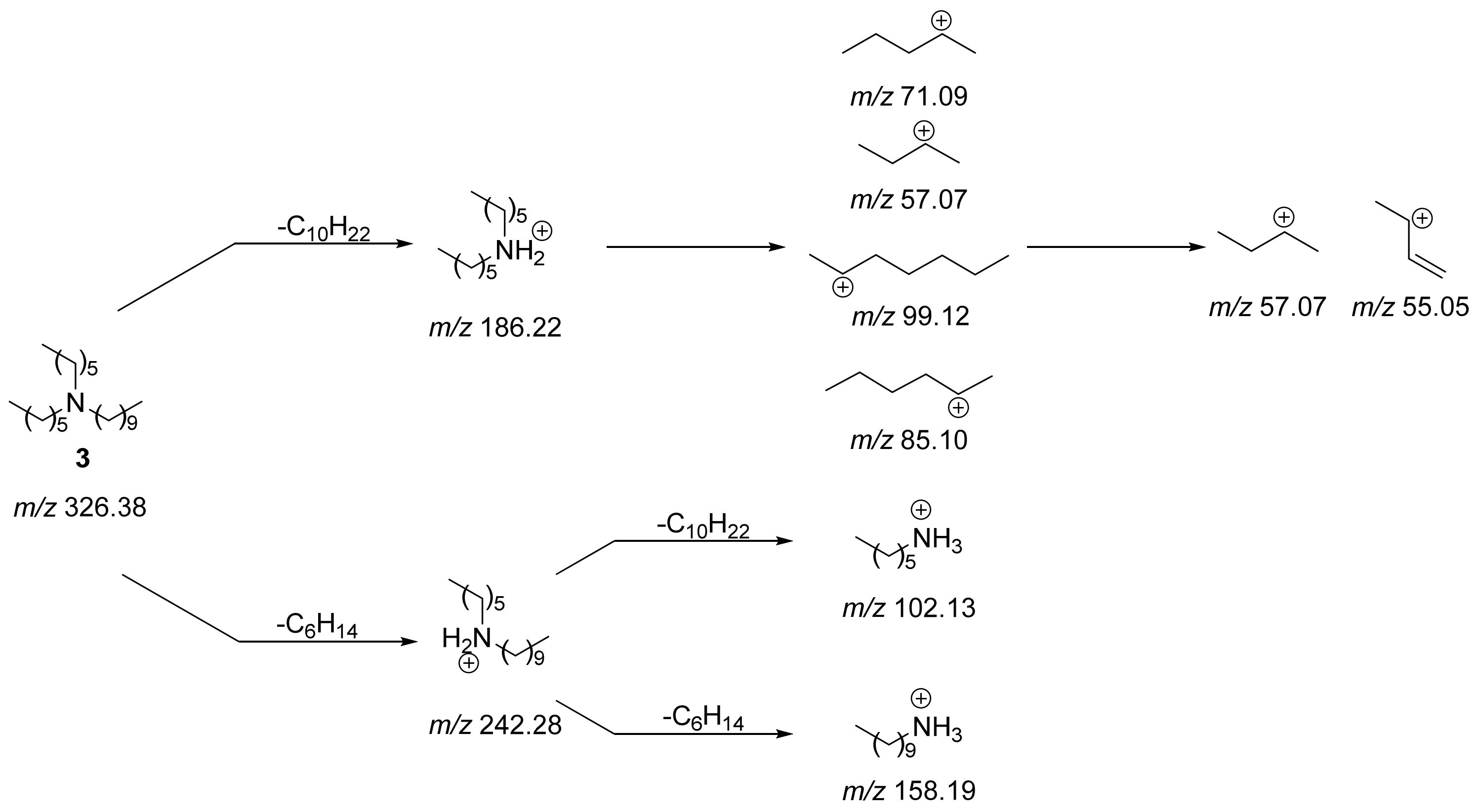

Figures S12 Key steps in the proposed MS/MS, MS^3 and MS^4 fragmentation pathway of the active component of AIMS sample 19033.

2.2 MS Analysis of AIMS Sample 20608.

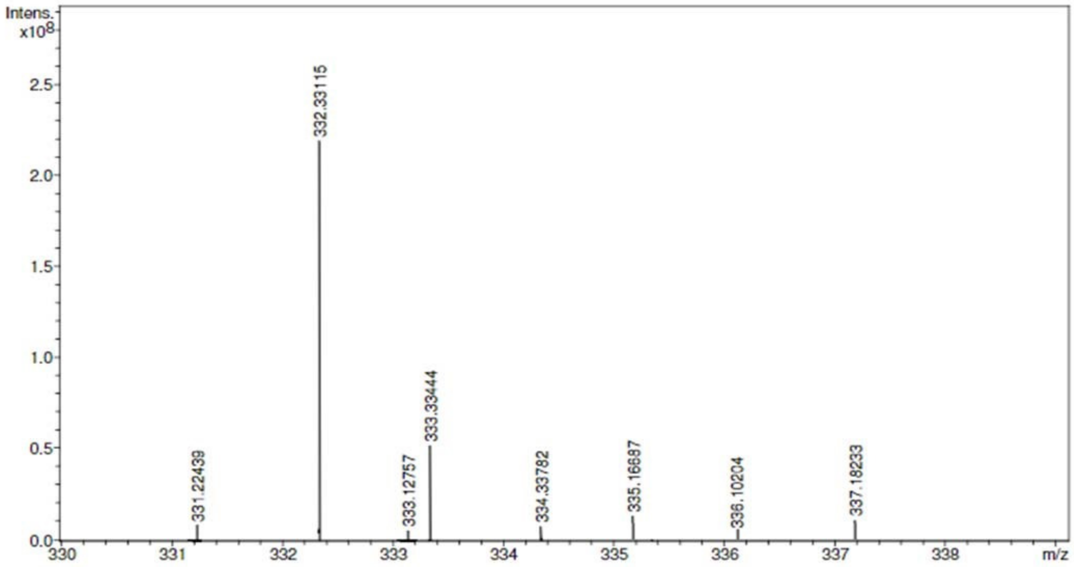

Figures S13 High resolution mass spectrum of the biologically active component of AIMS extract 20608.

By MS (Figures S13), accurate mass measurements for $[M+H]^+$ at *m/z* 332 (the strongest signal in this spectrum) returned an experimental accurate mass of 332.33115; theoretical (assuming $[CHNO+Na_{0-1}]^+$) for $C_{23}H_{42}N$ = 332.33118 (Δm = 0.03 ppm). No evidence of deuterium incorporation was observed (Table S3, isotopic fine structure around 333.33 *m/z* (data not shown)). MS/MS data revealed key fragment ions corresponding to neutral loss of C_7H_8 (rdbe = 4, potentially toluene) to form $[C_{16}H_{34}N]^+$ (rdbe = 0). Neutral loss of $[C_7H_7]$ suggests the presence of tropylium ion, a further indication of the presence of a tolyl moiety. MS/MS measurements of the fragment at *m/z* 259 showed fragmentation to $[C_9H_7]^+$ potentially 1-ethynyl-4-methylbenzene or 1H-inden-2-ylium (Figures S14). Taken together, these data suggest that the compound is a tertiary amine in which one substituent is either a methylphenyl group, with the methyl group in an undetermined position (*ortho*, *meta* or *para*), or a benzyl group. The other two substituent are saturated hydrocarbon chains, including a total of 16 carbon atoms. One of these chains may be as long as C_{10} (based on the largest fragments), with some branching on one or both chains likely present. Thus, the generalised structures **1** and **2** were proposed for the active agents in AIMS sample 20608.

Table S3 MS/MS, MS^3 and MS^4 data and assignments for AIMS sample 20608.

Parent ion peak	MS/MS or MS^3 pattern	Neutral loss (*m/z* or amu)	Daughter ion peak	Fragment form	Predictive structure
**332 $[C_{23}H_{42}N+H]^+$ (rdbe 4)**	→ 240 $[C_{16}H_{34}N]^+$ loss of (C_7H_8)	(C_7H_{10})	238	$[C_{16}H_{32}N]^+$ (rdbe 0)	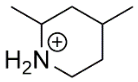 $[C_7H_{16}N]^+$
		$(C_{11}H_{25}N)$	69	$[C_5H_9]^+$ (rdbe 1)	
		$(C_{10}H_{23}N)$	83	$[C_6H_{11}]^+$ (rdbe 1)	
		$(C_9H_{21}N)$	97	$[C_7H_{13}]^+$ (rdbe 1)	
		$(C_8H_{19}N)$	111	$[C_8H_{15}]^+$ (rdbe 1)	
		(C_9H_{18})	114	$[C_7H_{16}N]^+$ (rdbe 1)	
	→ 240 → 97	(C_3H_6)	55	$[C_4H_7]^+$ (rdbe 1)	
	→ 240 → 114	$(C_{10}H_{20})$	100	$[C_6H_{14}N]^+$ (rdbe 1)	
	→ 259	$(C_{10}H_{24})$	115	$[C_9H_7]^+$ (rdbe 6)	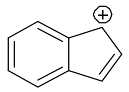 $[C_9H_7]^+$ or 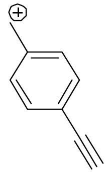
		(C_4H_{10})	201	$[C_{15}H_{21}]^+$ (rdbe 5)	
		(C_2H_{12}) or [M – C_3]	223	$[C_{17}H_{19}]^+$ (rdbe 8) or $[C_{16}H_{31}]^+$ (rdbe 1)	
		[M – 2H]	257	$[C_{19}H_{29}]^+$ (rdbe 6)	
		$(C_{16}H_{34}N)$	91	$[C_7H_7]^+$ (rdbe 5)	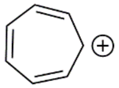

rdbe = ring or double bond equivalents

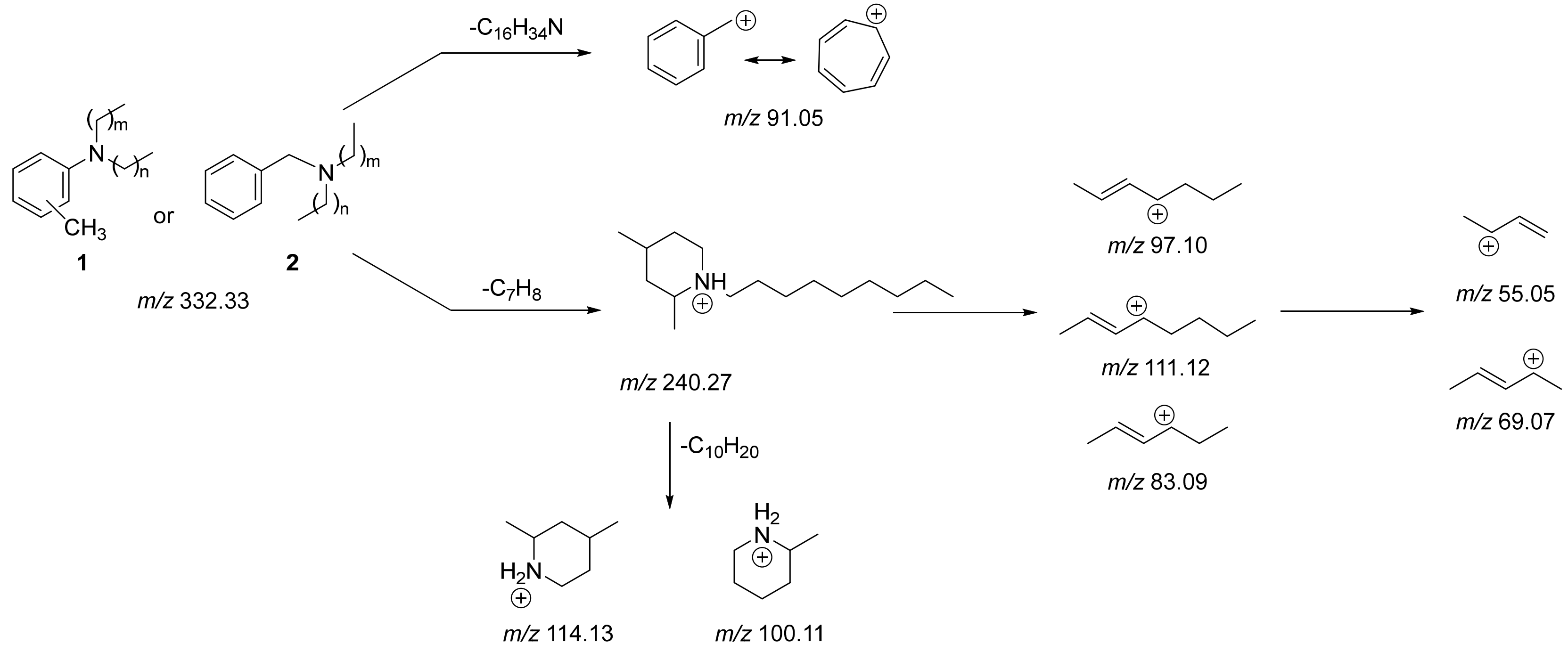

Figures S14 Proposed MS/MS, MS^3 and MS^4 fragmentations for compound 1/2 from AIMS sample 20608.

2.3 MS Analysis of AIMS Sample 26051.

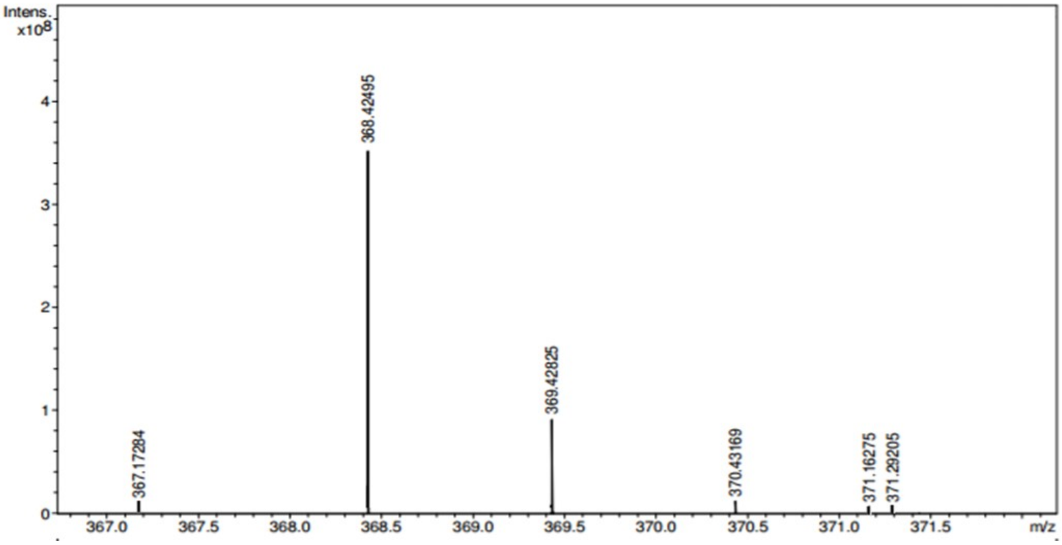

Figures S15 High resolution mass spectrum for the biologically active component of AIMS extract 26051.

Fractionation of the sample 26051 (Table S4 and Figures S4) afforded an active component of accurate MS (Figures S15) of experimental mass 368.42508; theoretical mass (assuming $[CHNO+Na_{0-1}]^+$) for $C_{25}H_{54}N$ = 368.42495; Δm = 0.13 ppm, RDBE = 0. MS/MS spectra showed five daughter ions giving fragments consistent with the presence of long hydrocarbon chains (Table S4), suggesting that this compound is fully saturated (0 RDBE) and is either a tertiary amine or quaternary amine salt, with one propyl chain and one of the other chains at least C_{10} in length (Figures S16).

Table S4 MS/MS and MS^3 data and assignments for AIMS samples 26051.

Parent ion	MS/MS or MS^3 pattern	Neutral loss (*m/z* or amu)	Daughter ion peak	Fragment form		Predictive structure
**368 $[C_{25}H_{54}N+H]^+$ (rdbe 0)**		C_3H_8	324	$[C_{22}H_{46}N]^+$	(1 rdbe)	Long hydrocarbon chain
		M-3H	365	$[C_{26}H_{53}]^+$ or $[C_{25}H_{49}O]^+$		
		NH_2+H_2	349	$[C_{25}H_{49}]^+$	(1 rdbe)	
		C_3H_9N	309	$[C_{22}H_{45}]^+$	(0 rdbe)	
		$C_5H_{15}N$	279	$[C_{20}H_{39}]^+$	(1 rdbe)	
	→ 324	C_3H_7N	267	$[C_{19}H_{39}]^+$	(0 rdbe)	
		$C_{12}H_{29}N$	137	$[C_{10}H_{17}]^+$	(2 rdbe)	
		$C_{13}H_{27}N$	123	$[C_9H_{15}]^+$	(2 rdbe)	
		$C_{14}H_{25}N$	109	$[C_8H_{13}]^+$	(2 rdbe)	
		$C_{15}H_{25}N$	97	$[C_7H_{13}]^+$	(1 rdbe)	
	→ 309	C_4H_{10}	251	$[C_{18}H_{35}]^+$	(1 rdbe)	

rdbe = ring or double bond equivalents

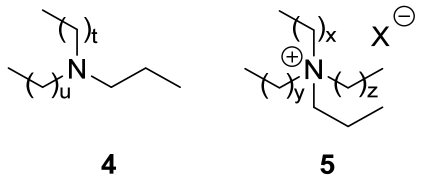

Figures S16 Proposed general structures for the tertiary amines isolated from 26051; (t + u) = 20; (x + y + z) =

2.4 MS Analysis of AIMS Sample 25663

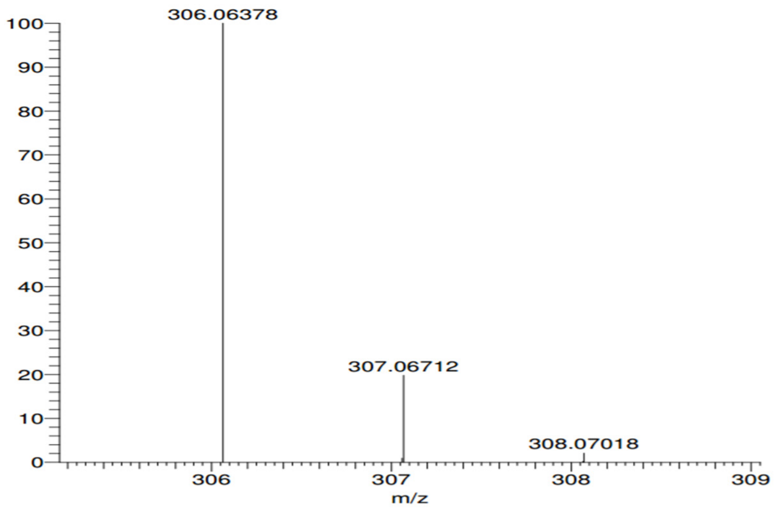

Figures S17 High resolution mass pattern for pure and biologically active fraction 44 from 25663.

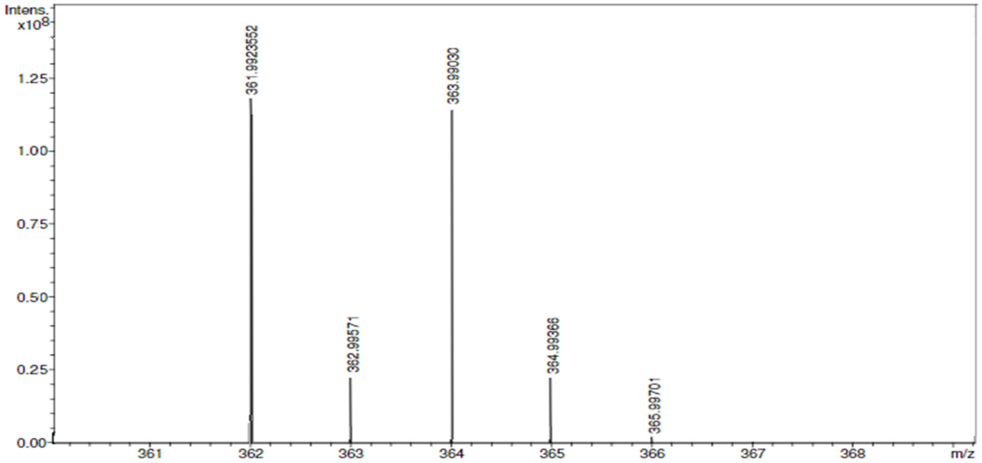

Figures S18 High resolution mass pattern for pure and biologically active fraction 58 from 25663.

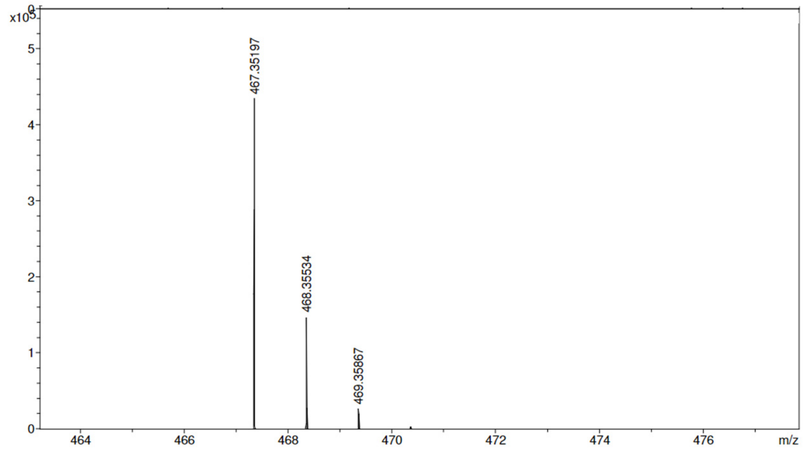

2.5 MS Analysis of AIMS Sample 26104

Figures S19 Low resolution atmospheric pressure chemical ionisation mass pattern for pure and biologically active fraction 69 from 26104.

2. NUCLEAR MAGNETIC RESONANCE SPECTROSCOPY DATA FOR NATURAL PRODUCTS.

3.1 NMR Analysis of AIMS Samples 25663

Table S5 ^{1}H (500 MHz) and ^{13}C (125 MHz) NMR spectroscopic data for compound 6.

Position	δH	δH, multiplicity (*J* in Hz)	COSY (nearest Hs)
**21**	9.20	d, 5.6	19
**17**	9.10	dd, 4.5, 1.7	16,15
**6**	9.00	dd, 8.0, 1.1	1,2,3
**19**	8.92	d, 5.6	21
**15**	8.63	dd, 7.9, 1.7	16,17
**3**	8.44	d, 8.0	1,2,6
**2**	8.09	ddd, 8.0, 7.2, 1.1	1,3,6
**1**	8.02	ddd, 8.0, 7.2, 1.1	2,3,6
**16**	7.80	dd, 7.9, 4.5	15,17

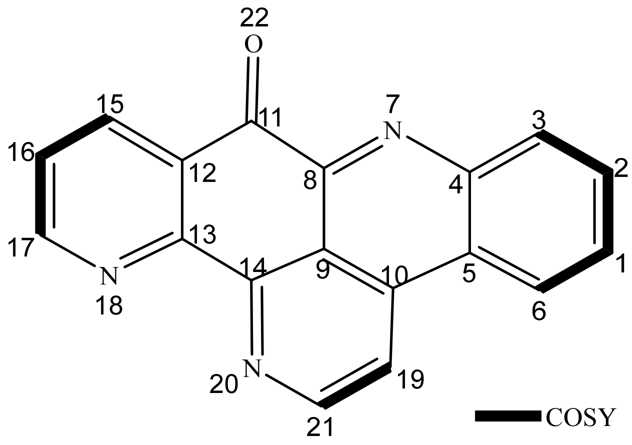

Figures S20 Structure of compound 6, Black bars indicate proton-proton correlations observed in the COSY NMR spectrum of this sample.

Table S6 ^{1}H (500 MHz) and ^{13}C (125 MHz) NMR spectroscopic data for compound 7.

Position	δH	δH, multiplicity (J in Hz)	COSY
**21**	9.25	d, 5.3	19
**17**	9.13	d, 4.3	16,15
**6**	8.98	d, 8.8	1,3
**19**	8.93	m	21
**15**	8.68	s	16,17
**3**	8.64	d, 7.6	1,6
**1**	8.19	d, 8.8	3,6
**16**	7.81	dd, 4.3, 7.6	15,17

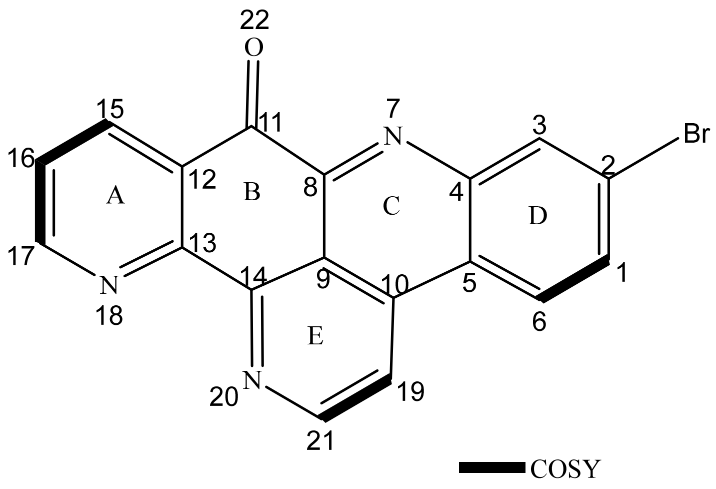

7

Figures S21 Structure of compound 7. Black bars indicate proton-proton correlations observed in the COSY NMR spectrum of this sample.

3.2 NMR Analysis of AIMS Samples 26104.

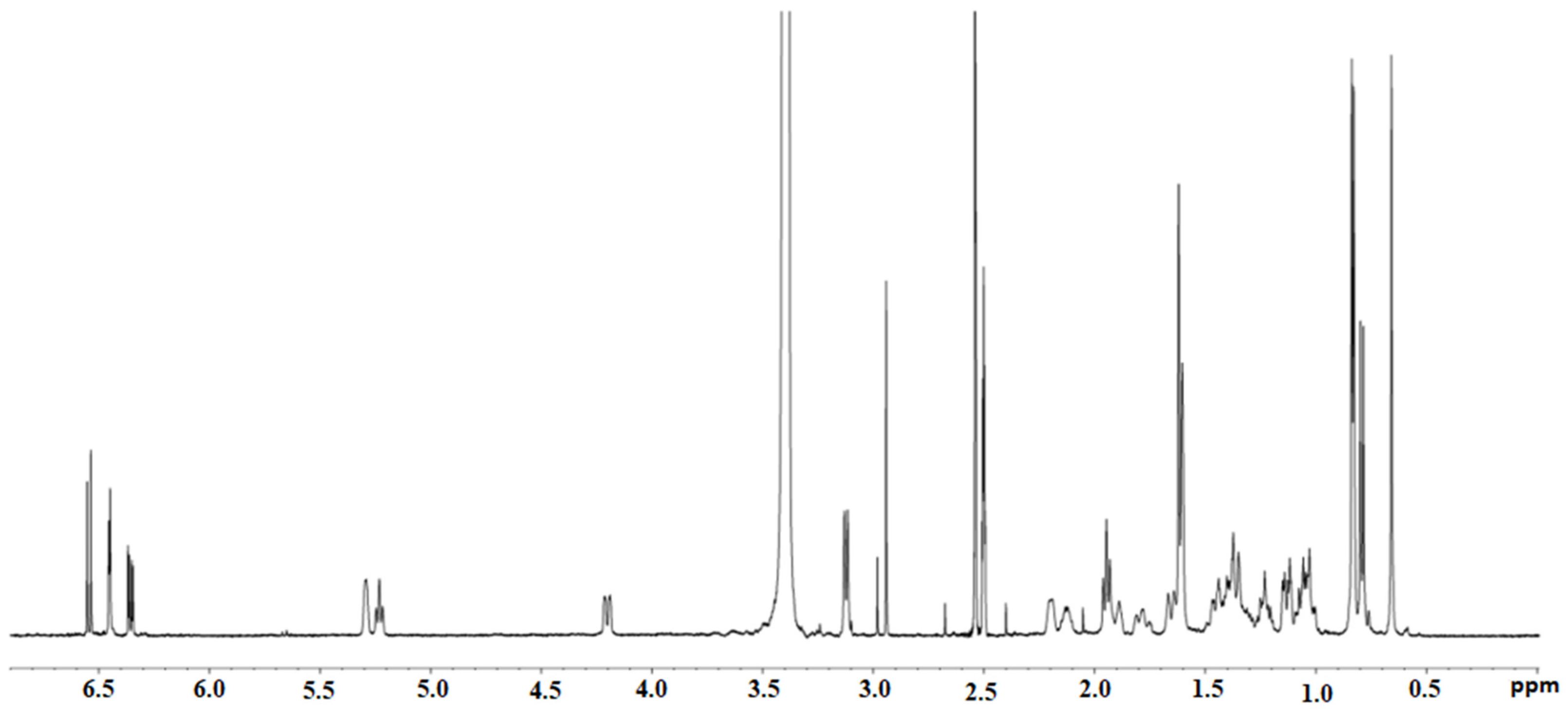

Figures S22 ^{1}H (500 MHz) NMR spectroscopic data for compound 8

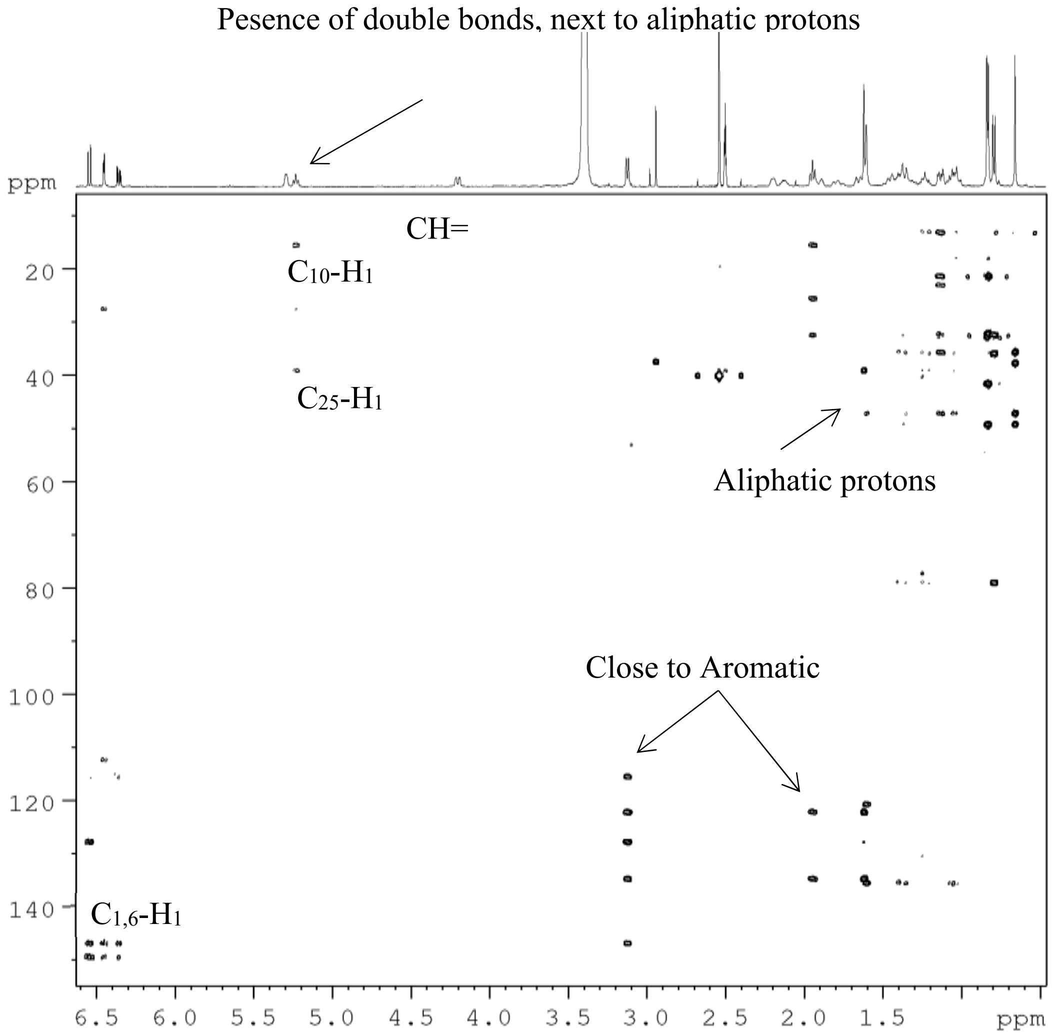
Pesence of double bonds, next to aliphatic protons
ppm
CH=
C10-H1
C25-H1
Aliphatic protons
Close to Aromatic
C1,6-H1
20
40
60
80
100
120
140
6.5
6.0
5.5
5.0
4.5
4.0
3.5
3.0
2.5
2.0
1.5
ppm

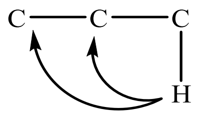
C
C
C
H

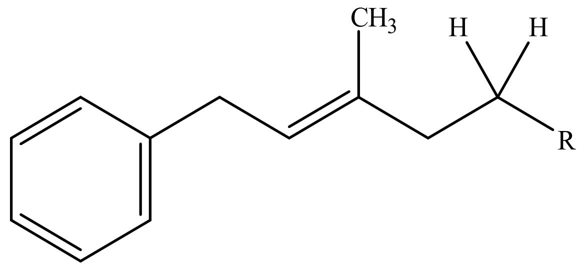

Figures S23 HMBC NMR spect

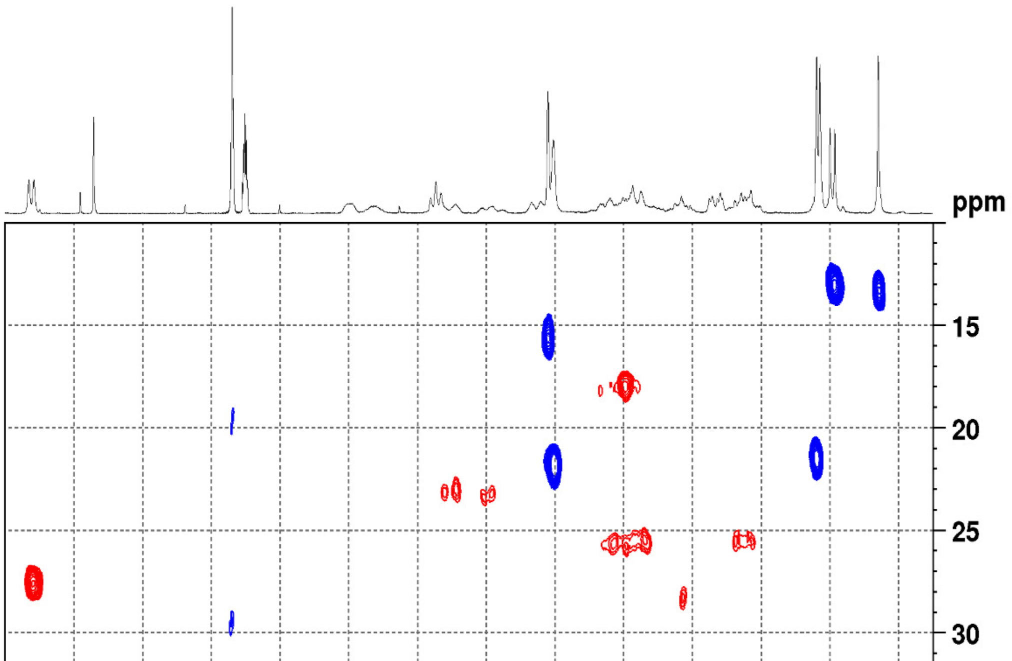

Key information [1]

C —— H

CH2

CH3 or CH

Bl dots: CH_3 or CH

Red dots: CH_2

Figures S24 HSQC NMR spectroscopic data for compound 8. Important correlations are highlighted.

Position	δH	δH, multiplicity (J in Hz)	HMBC	COSY
**8**	8.47	br s		
**7**	8.46	br s		
**6**	6.54	d, 8.4,	2, 1, 4	5
**3**	6.44	d, 3.2	9	4
**5**	6.35	dd, 3.2, 8.4		6, 4
**25**	5.29	m	31	24
**10**	5.22	t, 7.3	9,12,13	9
**18**	4.19	dm, 11.4	17,21	16,20
**9**	3.12	d, 7.3	3,10,2,11,1	10
**21**	2.19	m	32,31, 23	20
**16**	2.13	m	17	17,18
**13**	1.95	t, 7.4	10,12,15,14,2,11	14
**24**	1.91	m	23,25	23,25
**24´**	1.8	m		
**27**	1.66	dm, 13.1	32	28
**12**	1.62	s	10,11,13	
**31**	1.6	s	21, 25, 26	
**14**	1.42	m	13	13
**28**	1.41	m		27,29
**29**	1.37	m	34	28
**20**	1.34	m	22,21	21
**15**	1.21	m	13,17	
**23**	1.13	m	34, 33, 24, 32	24
**29´**	1.12	m		
**20´**	1.06	m		
**27´**	1.03	m		
**15´**	1.03	m		
**33**	0.84	s	23, 34, 30	

Table S7 ^{1}H (500 MH z) and ^{13}C (125 MHz) NMR spectroscopic data for compound 8.

**34**	0.83	s	29
**26**	0.78	d, 6.9	31
**32**	0.66	s	27, 22, 21, 23

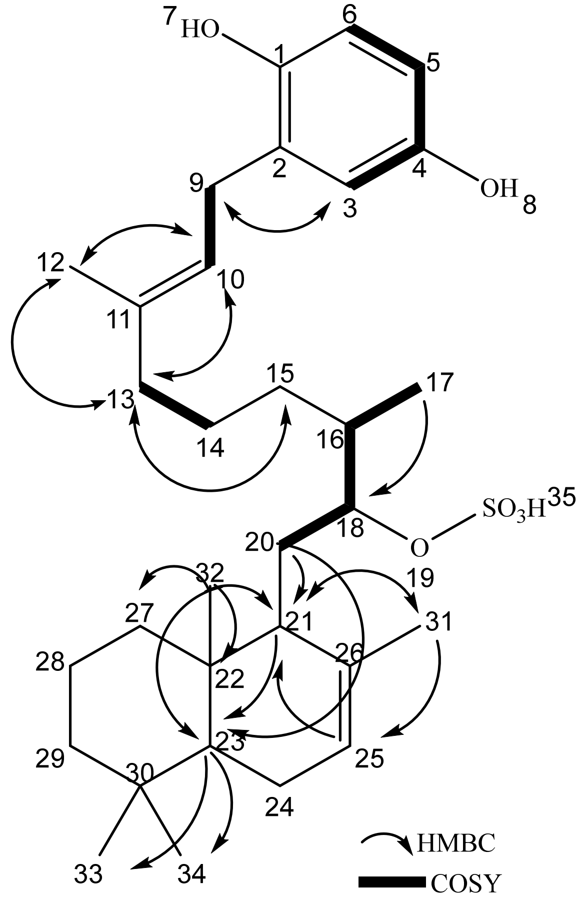

8

Figures S25 Structure of compound 8. Black bars and arrows indicate proton-proton and proton-carbon correlations observed in the COSY and HMBC NMR spectrum respectively, of this sample

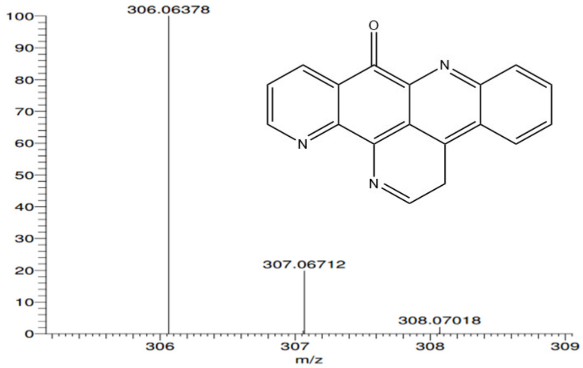

Compounds 6

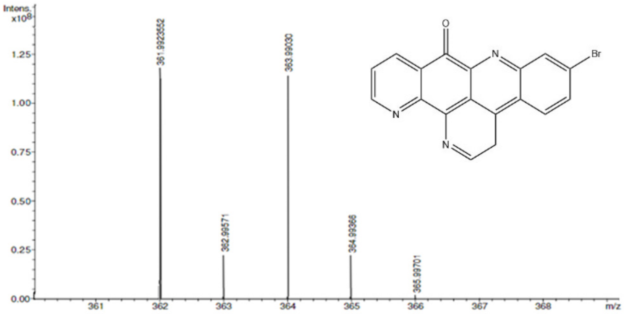

Compounds 7

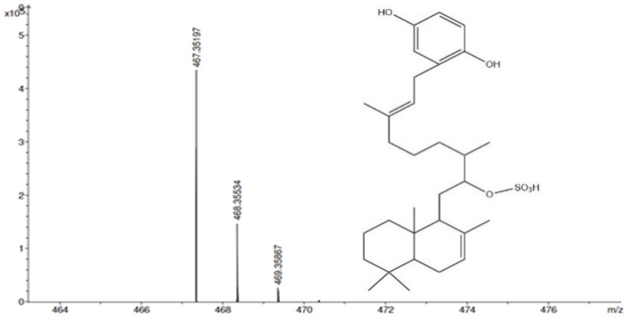

Compounds 8

Figures S26 Structures of compounds 6 ,7 and 8 with their mass patterns.

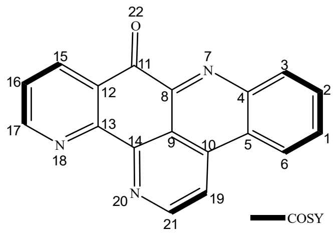

Compounds 6

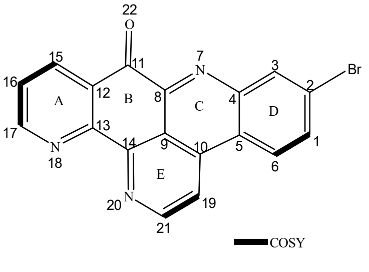

Compounds 7

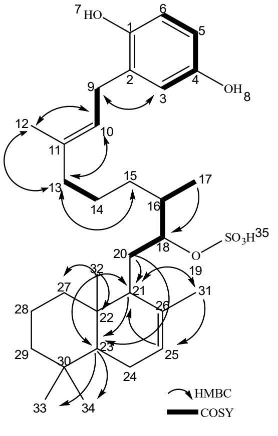

Compounds 8

Figures S27 Structures of compounds 6 ,7 and 8 with their NMR spectrum**.**
